# The increasing toll of adolescent cancer incidence in the US

**DOI:** 10.1371/journal.pone.0172986

**Published:** 2017-02-24

**Authors:** Jessica Burkhamer, David Kriebel, Richard Clapp

**Affiliations:** 1 Department of Public Health, University of Massachusetts, Lowell, Massachusetts, United States of America; 2 Lowell Center for Sustainable Production, University of Massachusetts Lowell, Lowell, Massachusetts, United States of America; Chang Gung Memorial Hospital Kaohsiung Branch, TAIWAN

## Abstract

Cancer incidence is rising among adolescents (“teens”). The causes of the increase are unknown but studying incidence patterns and trends may produce insights into etiology. Using data from the US National Cancer Institute’s Surveillance, Epidemiology, and End Results (SEER) program we described trends of cancer incidence among teens (15–19 year olds). We reviewed and summarized incidence patterns for histologic cancer groups and the most frequently diagnosed sites of cancer among teens during 2008–2012 reported by the SEER Cancer Statistics Review. We calculated annual incidence rates for the years 1975–2012 and used linear regression analysis to evaluate trends and calculate rates of change. Incidence for all sites combined increased annually by 0.67% for males and 0.62% for females during the period 1975 through 2012 –resulting in more than a 25% increase over 38 years. The biggest annual incidence increases occurred in non-Hodgkin lymphoma (NHL) (2.16% females; 1.38% males), thyroid cancer (2.12% females; 1.59% males), acute myeloid leukemia (AML) (1.73% females) and testicular cancer (1.55% males). Incidence rates for most histologic groups and sites showed steady long term increases over the 38 years of data. Despite improvements in survival, rising incidence trends mean growing numbers of young adults are undergoing painful and costly cancer treatments. A concerted research program is vital to investigate causes of steadily rising teen cancer rates.

## Introduction

Cancer incidence is rising among adolescents (“teens”) [1–3]. The causes of the increases are poorly understood but studying incidence patterns and trends may produce insights into etiology and suggest opportunities for prevention. However, with the exception of a small but growing body of research, study of cancer in teens has been relatively neglected in the US. The focus of cancer research has been on children and adults and research on cancer in teens and young adults has not kept pace [4]. Thus, the rise in teen cancer rates has not received the attention it deserves [3–5]. Although tremendous advances in cancer treatment have dramatically improved the survivability of cancer, even when “cured” cancer has devastating long-term effects including second malignancies and infertility [6]. Because teen cancer is relatively rare, large populations and long-term trends are needed for effective study. The goal of this article is to describe long-term-teen cancer trends in the US using data from the US National Cancer Institute’s Surveillance, Epidemiology, and End Results (SEER) program [7, 8]. To provide context for the trends, we first include a discussion of national teen cancer patterns presented in the tables 2.7, 32.1, 32.4, 32.7, and 32.17–32.19 of the SEER Cancer Statistics Review [1, 9].

**Fig 1:**
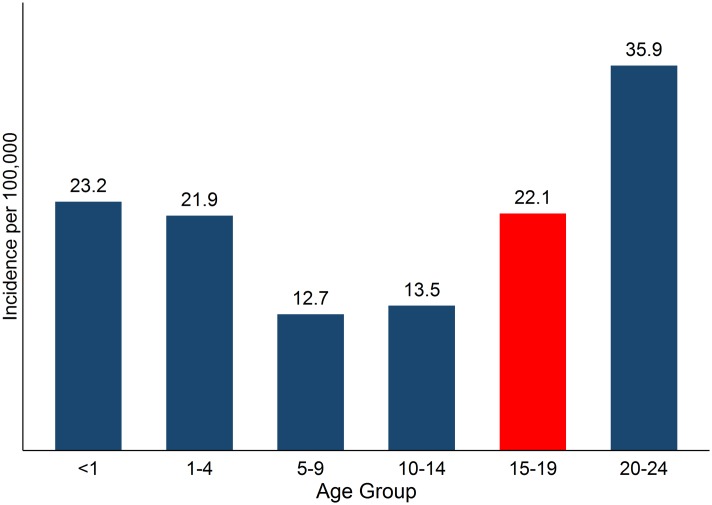
Age-specific incidence rates per 100,000, all sites combined, <1–24 years, 2008-2012^a^. ^a^Adapted from Howlader et al. 2015, Table 2.7.

The late childhood, adolescent, or teen years (15 to 19) may be considered a transitional phase between childhood and adulthood. In childhood, cancer incidence peaks in the first year of life and declines after that until 5–9 years of age. Cancer incidence rates start a notable upswing in the teen years (Fig 1) and incidence continues to increase with age throughout life [1, 3, 9]. Teens’ cancer experience differs from children and adults with regard to the kinds of cancers diagnosed. The most frequently diagnosed cancers among teens (males and females combined) are lymphomas, carcinomas, germ cell cancers, and leukemias. This list differs from the prevailing childhood cancers: leukemias, cancers of the central nervous system, and other pediatric and embryonal tumors, and also differ from adult cancers, which are dominated by carcinomas. In fact, the spectrum of common cancers shifts considerably from the teen years to older young adults (35–39 years) (S1 Fig) [1, 3, 5, 10].

It is widely accepted that morphology (histology) is more important in defining childhood cancers than topography (location), while topography is often thought to be more important for defining adult cancers, which are largely carcinomas. Teen cancers share characteristics from both of these age groups [5]. Barr and others developed a classification scheme tailored to suit the transitional characteristics of cancers among adolescents and young adults (AYA) aged 15–39 years, which include more histologic types than adult cancers and more carcinomas than childhood cancers but lack many of the embryonal tumors of childhood [2, 10]. This system has a flexible hierarchical structure where cancers are aggregated into nine groups that are subdivided into two or more sites or subgroups, which may be divided again as necessary. The SEER program uses an adapted version of this classification scheme to describe tumors in 15–29 year olds [1].

**Fig 2:**
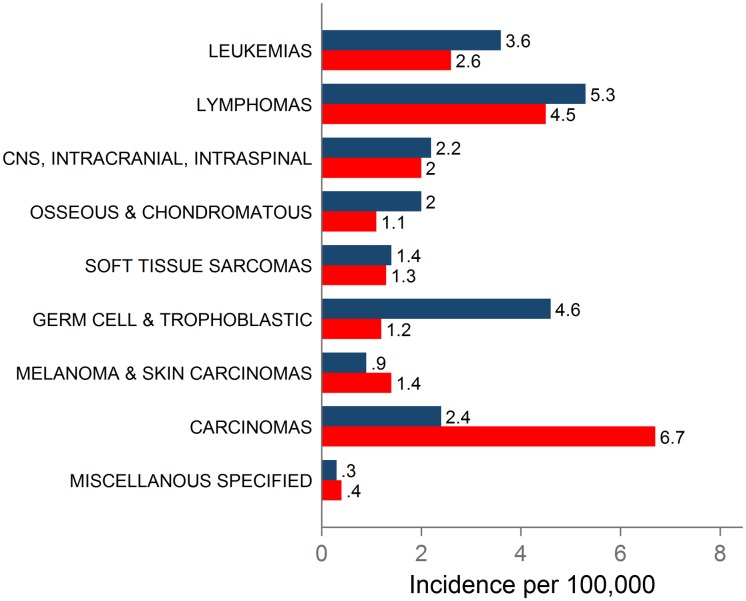
Histologic cancer group incidence rates, 2008–2012. Male incidence rates are shown with blue bars and female rates are shown by red bars^a^. ^a^Adapted from Howlader et al. 2015, Table 32.4.

According to the SEER Cancer Statistics Review, during 2008–2012, the incidence rate among teens for all sites combined was 22.9 per 100,000 for males and 21.3 for females (Table 1). The most frequent histologic diagnoses in males were lymphomas, germ cell and trophoblastic neoplasms, and leukemias. In females, the most frequent histologic diagnoses were carcinomas, lymphomas, and leukemias (Fig 2, Table 1). The most frequently diagnosed cancer sites in males were testicular cancer (germ cell and trophoblastic neoplasms of the gonads), Hodgkin lymphoma, NHL, acute lymphoid leukemia (ALL), astrocytoma, and osteosarcoma. In females, the most frequently diagnosed sites were thyroid cancer, Hodgkin lymphoma, melanoma, astrocytoma, NHL, and ALL (Table 1)[1].

**Table 1:** SEER cancer incidence per 100,000 and percent contribution, 2008–2012^a^.

DIAGNOSTIC GROUP/Site^b^	Male		Female	
	Rate^c^	% Contribution	Rate^c^	% Contribution
**TOTAL**	22.9	—	21.3	—
**LEUKEMIAS**	3.6	16%	2.6	12%
Acute lymphoid leukemia	2.2	10%	1.2	6%
Acute myeloid leukemia	0.9	4%	1.1	5%
**LYMPHOMAS**	5.3	23%	4.5	21%
Non-Hodgkin lymphoma	2.3	10%	1.2	6%
Hodgkin lymphoma	3	13%	3.3	15%
**CNS, INTRACRANIAL, INTRASPINAL**	2.2	10%	2	9%
Astrocytoma	1.2	5%	1.2	6%
**OSSEOUS & CHONDROMATOUS NEOPLASMS**	2	9%	1.1	5%
Osteosarcoma	1.0	4%	0.6	3%
**SOFT TISSUE SARCOMAS**	1.4	6%	1.3	6%
**GERM CELL AND TROPHOBLASTIC NEOPLASMS**	4.6	20%	1.2	6%
Gonadal (Testicular/Ovarian)	3.8	17%	1	5%
**MELANOMA AND SKIN CARCINOMAS**	0.9	4%	1.4	7%
Melanoma	0.9	4%	1.3	6%
**CARCINOMAS (excluding skin)**	2.4	10%	6.7	31%
Thyroid	0.9	4%	4.6	22%
**MISCELLANEOUS SPECIFIED NEOPLASMS, NOS**	0.3	1%	0.4	2%

^a^Adapted from Howlader et al. 2015, Table 32.4.

^b^ Classification scheme for tumors diagnosed in adolescents and young adults (Barr 2006).

^c.^ Rates for 2008–2012 and SEER 18 (San Francisco, Connecticut, Detroit, Hawaii, Iowa, New Mexico, Seattle, Utah, Atlanta, San Jose-Monterey, Los Angeles, Alaska Native Registry, Rural Georgia, California, Kentucky, Louisiana, New Jersey and Georgia).

A closer look at the makeup of the nine cancer groups shows that the groups defined as melanoma and skin carcinomas, germ cell cancers and trophoblastic neoplasms, and carcinomas were each dominated by a single cancer site (Table 1). Melanoma accounts for nearly all diagnoses in the melanoma and skin carcinomas group (100% for males and 93% for females). Germ cell cancers are primarily gonadal cancers, testicular cancer in males (83%) and ovarian cancer in females (83%). Thyroid cancer makes up the bulk of carcinomas in females (69%) and a large percentage of carcinomas in males (38%) [1].

As the lists of top cancer sites suggest, there are some differences between the patterns of cancer incidence for males and females (Fig 2). Generally, females tend to have slightly lower incidence rates than males. There are large differences between males and females in the incidence of germ cell cancers and carcinomas and moderate differences in the incidence of osseous and chondromatous neoplasms, melanoma and skin carcinomas, and leukemias. During 2008–2012, the incidence rate of germ cell and trophoblastic neoplasms of the gonads in males (testicular cancer) was nearly four times that of females (ovarian cancer), the rate of thyroid cancer in females was five times that of males, the rate of osseous and chondromatous neoplasms was 82% higher in males, the rate of melanoma and skin carcinomas was 56% higher in females, and the rate of leukemias was 38% higher in males [1].

After reviewing the SEER Cancer Statistics Review, we selected the most frequently diagnosed cancer sites among teens in the US as identified by Howlader (1) and others for trend analysis, focusing on direction and differences between males and females.

## Methods

Cancer sites were analyzed using the adapted classification scheme for AYAs used by the SEER program [1]. The classification scheme is based on the system developed by Barr and others to describe tumors in 15–29 year olds [11]. The adapted classification scheme for AYAs used by SEER is restricted to malignant tumors and updated based on the International Classification of Diseases for Oncology’s third edition of codes (ICD-O-3) and the World Health Organization (WHO) system of hematopoietic codes [1, 12, 13].

We describe and evaluate long-term trends for the nine AYA cancer groups and the most frequently diagnosed sites of cancer among teens (15–19 year olds) during 2008–2012 (the most recent five-year period rather than a single year was used to have more stable estimates of rates), based on the 2015 Cancer Statistics Review published by the SEER program and SEER incidence and population data [1]. We obtained cancer incidence data for the SEER 9 registries, which include Atlanta, Connecticut, Detroit, Hawaii, Iowa, New Mexico, San Francisco-Oakland, Seattle-Puget Sound, and Utah. Data are available for seven of the nine registries from 1973 through 2012; however, data from Atlanta and Seattle-Puget Sound are available starting in 1975. The SEER 9 registries cover about 9% of the US population and the population covered is comparable to the general US population with regard to race and ethnicity [14]. Trends observed in the SEER 9 registries are often considered to be representative of the general US population.

To evaluate trends in teen cancers, age-group specific (15–19 years) annual incidence rates were calculated for the years 1975–2012 and linear regression analysis was used to evaluate the direction of trends and calculate annual and total percent change. Linear regression has an intuitive and straightforward interpretation for examining the linear component of long-term time trends, and can be useful when making comparisons among a large number of different data sets, as in this article. Analyses of SEER data often use join point analysis [1, 15] instead of linear regression. Although the former method does not assume linearity and is in that sense a more flexible method, we believe that linear regression facilitates comparisons among trends because it provides a consistent and straightforward estimate of long term trends in incidence.

**Fig 3:**
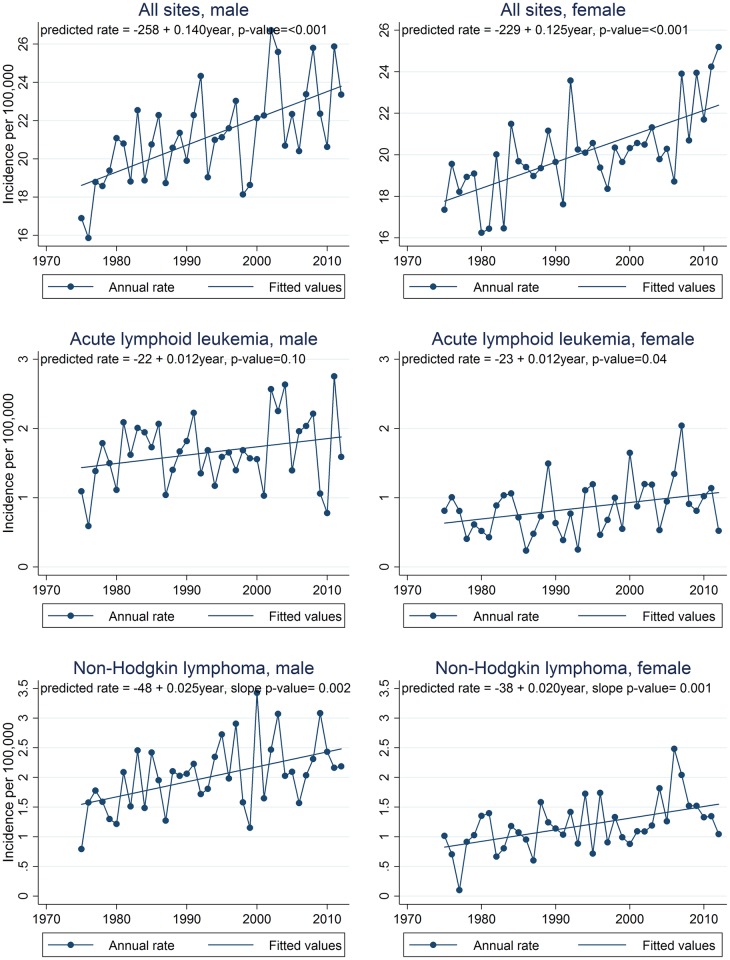
Annual incidence trends for all sites combined, acute lymphoid leukemia, and Non-Hodgkin lymphoma, 1975–2012, SEER 9 registries. Annual rates are shown by connected points and the least squares regression line is shown as a straight line. Regression equations and p-values for trend are also shown. SEER 9 registries include Atlanta, Connecticut, Detroit, Hawaii, Iowa, New Mexico, San Francisco-Oakland, Seattle-Puget Sound, and Utah.

**Fig 4:**
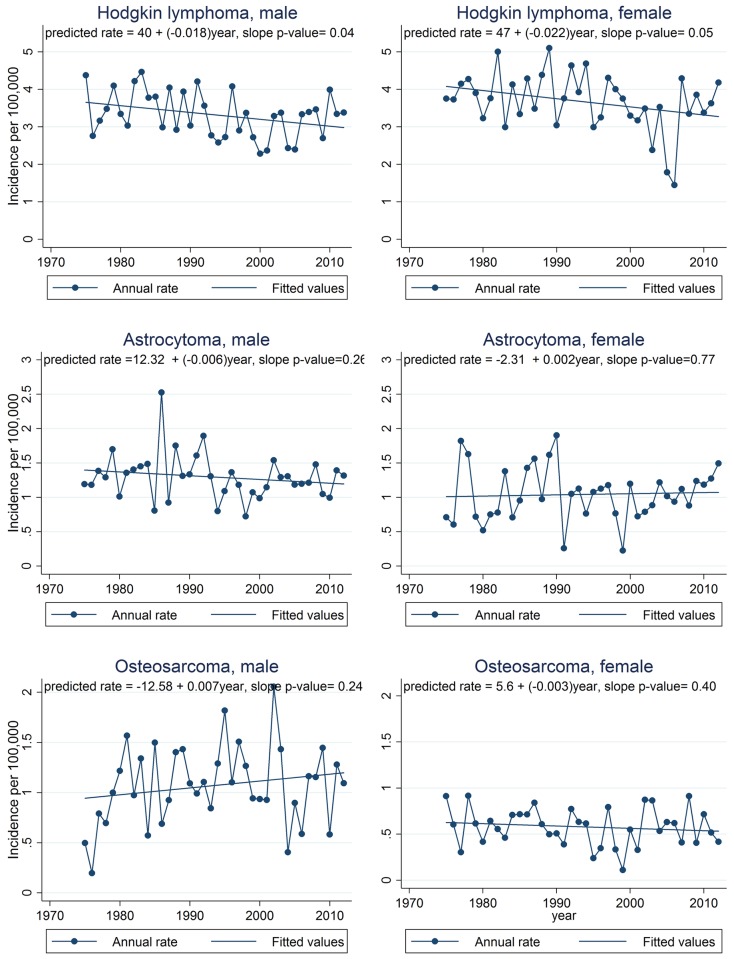
Annual incidence trends for Hodgkin lymphoma, astrocytoma, and osteosarcoma, 1975–2012, SEER 9 registries. Annual rates are shown by connected points and the least squares regression line is shown as a straight line. Regression equations and p-values for trend are also shown. SEER 9 registries include Atlanta, Connecticut, Detroit, Hawaii, Iowa, New Mexico, San Francisco-Oakland, Seattle-Puget Sound, and Utah.

**Fig 5:**
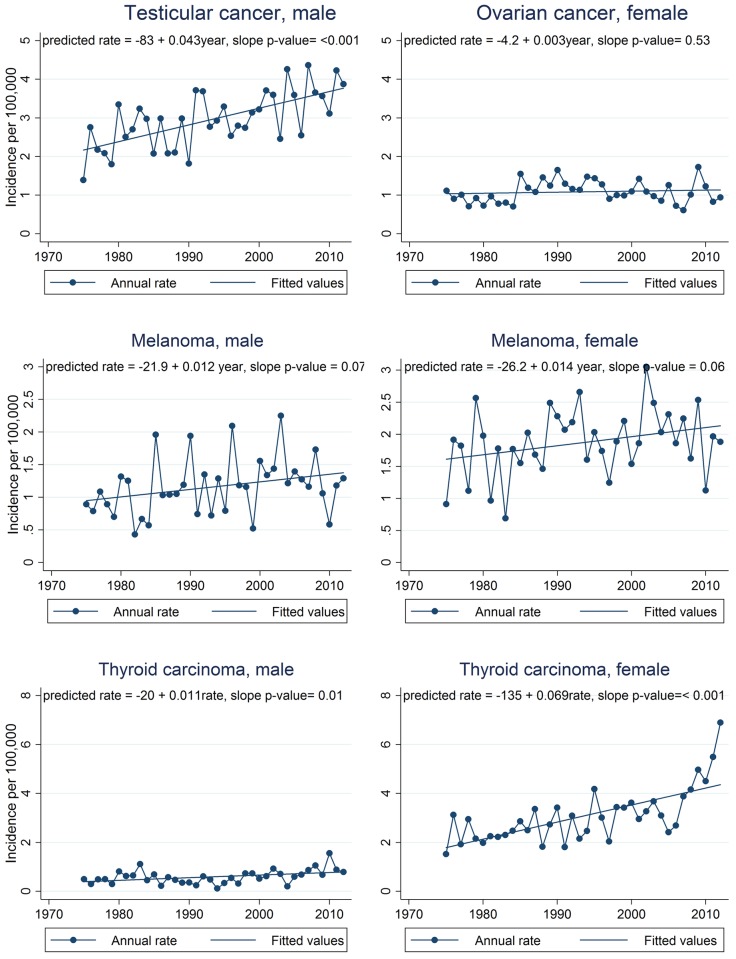
Annual incidence trends for testicular cancer, melanoma, and thyroid cancer, 1975–2012, SEER 9 registries. Annual rates are shown by connected points and the least squares regression line is shown as a straight line. Regression equations and p-values for trend are also shown. SEER 9 registries include Atlanta, Connecticut, Detroit, Hawaii, Iowa, New Mexico, San Francisco-Oakland, Seattle-Puget Sound, and Utah.

Graphs of observed annual incidence rates for the most common cancer sites are presented in Figs 3, 4 and 5 along with linear trend lines, their regression equations, and p-values for trend. Graphs for cancer sites not presented in Figs 3, 4 and 5 are available in supporting information (S2–S4 Figs). For additional information in interpreting the trends by site, S1 Table provides a summary of the annual numbers of cases by cancer site. The assumption of linearity in cancer incidence trends over the full 38 year period for which SEER 9 data are available was evaluated by using the p-value for the slope in linear regression models. For each regression, the p-value from the t-test of the slope was reported. The t-test evaluates the null hypothesis that the derived slope could be obtained by chance if there was no linear trend. Thus a small p-value is evidence that there is an important linear component to the long term trend. We defined p < 0.05 as statistically significant. Total percent change in the incidence rate from 1975 through 2012 (38 years) was calculated using the fitted rates from the regression analysis and comparing the predicted rates in the first and last years of the period expressed as a percent of the 1975 rate. Cancers in teens are rare and thus even the national incidence rates fluctuate quite a bit from year to year. Thus, using the fitted rates to calculate total percent change results in more stable estimates of trends rather than simply comparing observed first and last year rates.

Because we used linear regression instead of join point analysis, we calculate the annual percent change (APC) instead of the average annual percent change (AAPC), which is calculated for analyses using the joint point method. The annual percent change in the incidence rate was calculated using the method used by the SEER program [16]. Briefly, the APC was calculated by fitting a least squares regression line using calendar year as the predictor and the natural log of annual rates as the dependent variable. The standard error for the APC was obtained from the linear regression model and used to calculate 95% confidence intervals:

$$APC=100\times \left(e^{slope}-1\right)$$

$$CI=\left(e^{\left(slope\pm \left(Tvalue\times SEslope\right)\right)}-1\right)\times 100$$

## Results

The assumption of linearity in cancer incidence trends over the full 38 year period for which SEER 9 data are available was evaluated by graph examination and by assessing the p-value for the slope in linear regression models. Graphs of annual incidence rates for selected cancer sites are presented in Figs 3, 4 and 5 along with linear trend lines, their regression equations, and p-values for trend. Graphs for cancer sites not presented in Figs 3, 4 and 5 are available in additional file (S2–S4 Figs).

**Fig 6:**
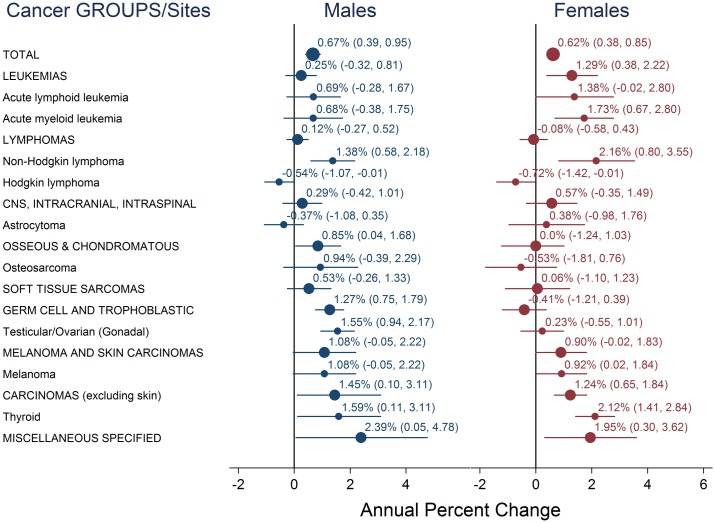
Annual percent change for all sites combined, histologic cancer groups, and nine sites, 1975–2012. Graphs showing annual percent change for all sites combined (large circles, labeled in all capital letters), histologic cancer groups (medium circles, labeled in all capital letters), and nine cancer sites (small circles). The 95% confidence intervals are shown as error bars extending from the markers.

Trend analysis of SEER 9 data indicated that teen cancer incidence overall increased during the period 1975 through 2012. During this time period, incidence for all sites combined increased annually by 0.67% for males and 0.62% for females (Figs 3–6). These rate increases mean that for every 100,000 teens, an estimated 5.2 additional males and 4.6 additional females were diagnosed in 2012 compared to 1975, increases of about 28% and 26%, respectively, over the entire 38 year period. Of the estimated 4,900 teens diagnosed with cancer in the US in 2012, more than 1,000 diagnoses could be attributed to these long-term incidence rate increases (Table 2). Trends were positive for all cancer groups in males and for all groups except lymphomas, germ cell tumors, and osseous and chondromatous neoplasms in females. Within these groups, incidence rate trends were positive for all sites evaluated, except for Hodgkin lymphoma and astrocytoma in males and Hodgkin lymphoma and osteosarcoma in females. While incidence of most cancer sites and subgroups evaluated increased, trends for Hodgkin lymphoma were negative for males and females, astrocytoma decreased in males, and osteosarcoma decreased in females.

**Table 2:** Total incidence rate change 1975–2012 and estimated change in diagnoses nationally.

Diagnostic GROUP/Site^a^	Male						Female						Total					
	Change in rates^b^	Change in number of diagnoses	1975 Rate	Estimated 2012 diagnoses using 1975 rate	2012 Rate	Estimated 2012 diagnoses	Change in rates	Change in number of diagnoses	1975 Rate	Estimated 2012 diagnoses using 1975 rate	2012 Rate	Estimated 2012 diagnoses	Change in rates	Change in number of diagnoses	1975 Rate	Estimated 2012 diagnoses using 1975 rate	2012 Rate	Estimated 2012 diagnoses
**TOTAL**	5.18	568	18.62	2,040	23.80	2,608	4.62	480	17.77	1,847	**22.39**	**2,328**	**4.91**	**1,050**	**18.20**	**3,887**	**23.12**	**4,936**
**LEUKEMIAS**	0.35	38	2.67	292	3.02	331	0.94	98	1.54	160	**2.48**	**258**	**0.65**	**138**	**2.11**	**451**	**2.76**	**589**
Acute lymphoid leukemia	0.44	48	1.44	158	1.88	206	0.44	46	0.64	66	1.08	112	0.87	185	0.97	208	1.84	393
Acute myeloid leukemia	0.19	21	0.74	81	0.93	102	0.47	49	0.58	60	1.05	109	0.37	80	0.64	136	1.01	216
**LYMPHOMAS**	0.26	28	5.21	571	5.46	599	-0.08	-8	4.90	510	**4.82**	**501**	**0.10**	**20**	**5.06**	**1080**	**5.15**	**1,100**
Non-Hodgkin lymphoma	0.93	102	1.55	170	2.48	272	0.72	75	0.83	86	1.55	161	0.68	145	1.21	259	1.89	404
Hodgkin lymphoma	-0.68	-75	3.66	401	2.98	327	-0.81	-84	4.08	424	3.27	340	-0.98	-209	3.89	831	2.91	622
**CNS, INTRACRANIAL, INTRASPINAL**	0.21	23	2.14	235	2.35	258	0.31	32	1.57	163	**1.88**	**195**	**0.26**	**56**	**1.86**	**397**	**2.12**	**453**
Astrocytoma	-0.20	-22	1.40	153	1.19	131	0.06	6	1.01	105	1.07	112	-0.07	-15	1.21	258	1.14	243
**OSSEOUS & CHONDROMATOUS NEOPLASMS**	0.6	66	1.75	192	2.35	258	0.01	1	1.11	115	**1.12**	**117**	**0.32**	**68**	**1.43**	**306**	**1.75**	**375**
Osteosarcoma	0.25	27	0.94	104	1.20	131	-0.09	-9	0.63	65	0.53	55	0.09	19	0.79	170	0.88	188
**SOFT TISSUE SARCOMAS**	0.23	25	1.43	157	1.66	182	0.11	11	1.39	144	**1.50**	**156**	**0.17**	**37**	**1.41**	**301**	**1.58**	**338**
**GERM CELL AND TROPHOBLASTIC NEOPLASMS**	1.59	174	2.77	304	4.37	479	-0.14	-15	1.43	149	**1.29**	**134**	**0.76**	**163**	**2.11**	**451**	**2.87**	**613**
Testicular/Ovarian	1.6	175	2.17	238	3.77	413	0.1	10	1.04	108	1.14	118	0.89	189	1.61	344	2.50	533
**MELANOMA AND SKIN CARCINOMAS**	0.45	49	0.96	105	1.41	154	0.5	52	1.63	170	**2.13**	**222**	**0.47**	**101**	**1.29**	**275**	**1.76**	**376**
Melanoma	0.43	47	0.95	104	1.38	151	0.52	54	1.61	168	2.13	222	0.11	23	1.36	289	1.46	312
**CARCINOMAS (excluding skin)**	1.05	115	1.44	158	2.49	273	2.62	272	3.83	398	**6.45**	**671**	**1.79**	**383**	**2.62**	**560**	**4.41**	**943**
Thyroid	0.39	43	0.40	44	0.79	87	2.57	267	1.79	186	4.36	453	1.12	239	1.16	247	2.27	486
**MISCELLANEOUS SPECIFIED NEOPLASMS, NOS**	0.44	48	0.17	19	0.62	68	0.31	32	0.29	30	**0.60**	**62**	**0.35**	**74**	**0.25**	**54**	**0.60**	**128**

^a^Classification scheme for tumors diagnosed in adolescents and young adults (Barr 2006).

^b^Change in predicted rates calculated from SEER 9 registries, 1975–2012.

NHL, thyroid cancer, and AML in females had the highest annual percent increases: 2.16%, 2.12%, and 1.73%; followed by thyroid cancer, testicular cancer, and NHL in males: 1.59%, 1.55%, 1.38%. APCs for melanoma and ALL in females and osteosarcoma in males were also among the largest observed. Among these seven cancer sites, only the trends for osteosarcoma in males and melanoma in males and females had p-values slightly above the 0.05 significance level (Table 3). In contrast to these increasing trends, Hodgkin lymphoma had an annual *decrease* of 0.54% and 0.72% in males and females, astrocytoma decreased by 0.43% annually in males, and osteosarcoma decreased 0.53% annually in females. However, these weakly negative trends were not statistically significant.

**Table 3:** Incidence rate trend slopes.

Diagnostic GROUP/Site^a^	Male			Female		
	slope	95% CI^b^	p-value	slope	95% CI^b^	p-value
**TOTAL**	0.14	(0.081, 0.199)	0.00	0.125	(0.077, 0.173)	0.00
**LEUKEMIAS**	0.009	(-0.007, 0.026)	0.26	0.025	(0.008, 0.043)	0.01
Acute lymphoid leukemia	0.012	(-0.003, 0.027)	0.10	0.012	(0.001, 0.023)	0.04
Acute myeloid leukemia	0.005	(-0.004, 0.014)	0.25	0.013	(0.005, 0.020)	0.00
**LYMPHOMAS**	0.007	(-0.014, 0.028)	0.51	-0.002	(-0.016, 0.022)	0.85
Non-Hodgkin lymphoma	0.025	(0.010, 0.040)	0.00	0.02	(0.008, 0.031)	0.00
Hodgkin lymphoma	-0.018	(-0.036, -0.001)	0.05	-0.022	(-0.044, -0.000)	0.04
**CNS, INTRACRANIAL, INTRASPINAL**	0.006	(-0.012, 0.023)	0.51	0.008	(-0.006, 0.023)	0.24
Astrocytoma	-0.006	(-0.015, -0.004)	0.26	0.002	(-0.010, 0.013)	0.77
**OSSEOUS & CHONDROMATOUS NEOPLASMS**	0.016	(0.002, 0.031)	0.03	0	(-0.012, 0.012)	0.96
Osteosarcoma	0.007	(-0.005, 0.019)	0.243	-0.003	(-0.009, 0.004)	0.4
**SOFT TISSUE SARCOMAS**	0.006	(-0.006, 0.018)	0.29	0.003	(-0.012, 0.018)	0.69
**GERM CELL AND TROPHOBLASTIC NEOPLASMS**	0.043	(0.026, 0.060)	0.00	-0.004	(-0.015, 0.007)	0.48
Gonadal (Testicular/Ovarian)	0.043	(0.026, 0.060)	0.00	0.003	(-0.006, 0.011)	0.53
**MELANOMA AND SKIN CARCINOMAS**	0.012	(0.000, 0.025)	0.05	0.014	(-0.002, 0.029)	0.08
Melanoma	0.012	(-0.001, 0.024)	0.07	0.014	(-0.001, 0.290)	0.06
**CARCINOMAS (excluding skin)**	0.028	(0.015, 0.042)	0.00	0.071	(0.037, 0.104)	0.00
Thyroid	0.011	(0.003, 0.019)	0.01	0.069	(0.045, 0.093)	0.00
**MISCELLANEOUS SPECIFIED NEOPLASMS, NOS**	0.012	(0.004, 0.020)	0.00	0.008	(0.002, 0.015)	0.01

^a^Classification scheme for tumors diagnosed in adolescents and young adults (Barr 2006).

^b^95% CI—95% confidence interval.

Incidence rate trends were similar in males and females for most cancers, but differed conspicuously for germ cell and trophoblastic neoplasm of the gonads (testicular and ovarian cancer) and thyroid cancer. The ovarian cancer trend was essentially flat and was not statistically significant while the testicular cancer trend was about an order of magnitude larger. Testicular cancer increased 1.55% annually, the predicted rate increasing from 2.17 to 3.77 per 100,000—a total increase of 74% over the last 38 years. While for thyroid cancer, the slope for females (0.069) was about six times higher than for males (0.011). The APC for females was 2.12% and was 1.59% for males. Due to the rate increases an estimated 267 additional females and 43 additional males were diagnosed with thyroid cancer nationally in 2012 compared to what would have been expected if the rate had remained at the 1975 level.

NHL incidence rose steeply in both sexes. The APC for females was higher than males (2.16% vs. 1.38%). In contrast, Hodgkin lymphoma incidence rates dropped annually, by 0.54% for males and 0.72% for females. Decreases in Hodgkin lymphoma were similar for males and females resulting in about 75 fewer diagnoses in males and 84 fewer diagnoses in females in 2012.

The annual rate increase for leukemias in females was five times that of males. Female rates increased 1.29% annually for a total increase of 61% from 1975 to 2012. Rates of leukemias in males increased more modestly, at 0.25% per year for a total increase of 13%. The trends for ALL, AML, and leukemias as a group in males were not statistically significant. APCs for ALL and AML in females were more than double those in males.

Trends for melanoma also increased for both males and females, with increases of 1.08% and 0.92% for males and females respectively. An estimated 151 males and 222 females were diagnosed with melanoma nationally in 2012. About a third of the diagnoses in males and a quarter of female diagnoses could be accounted for by the long-term rise in incidence rates.

## Discussion

Our analyses showed that among 15–19 year old teens in the US, overall cancer incidence has been increasing from the mid-1970s to 2012. Increases in incidence rates of all cancers combined in US teens have also been noted by Howlader and others in the Cancer Statistics Review, as well as by Bleyer and colleagues [1, 3]. We believe however that this paper is the first to systematically calculate and graphically present the long term trends in cancer incidence for this age group. The reasons for these rising teen cancer rates are not known. Some cancer researchers argue that improved technology, detection methods, and diagnoses account for this rise in incidence [17], while others argue that if this were the case, one would expect to see cancer incidence rates flattening, which has not yet occurred. In fact, incidence rates for most cancer groups and six of the nine most common cancer sites have been increasing reasonably linearly since 1975.

There is good evidence that thyroid cancer diagnosis in adults has been increasing at least in part because of increased screening [18]. One might assume that this phenomenon is also occurring among teens, but it is difficult to see how increased screening would explain the marked sex difference we observed—an obviously increasing incidence trend in females and a stable to slightly increasing trend in males. This sex difference was previously reported by Bleyer and others (2006) for the period 1975–2000 and our evaluation shows that this trend has continued through 2012.

For many of the most common teen cancers, known risk factors are unlikely to account for the majority of cases [3]. Childhood cancers, including teen cancers, are often thought of as being sporadic, rising from new mutations by chance. If these cancers are truly sporadic, why would their incidence increase over time? The answer to this question is unknown but the presence of yet to be confirmed risk factors seems likely.

Hodgkin lymphoma is the only cancer type examined here with a statistically significant decreasing trend. Environmental exposure opportunities have long been thought to influence incidence of Hodgkin lymphoma because in economically developed countries incidence peaks in young adults and older adults and the nodular sclerosis subtype dominates, while in developing countries, incidence peaks occur in children (particularly among boys) and older adults and the mixed cellularity and lymphocyte-depleted subtypes are most common. Epstein-Barr virus infection and high socio-economic status are associated with increased risk of Hodgkin lymphoma, thus it has been suggested that differences in exposure opportunities, such as delayed EBV infection in wealthier countries, explain the distinct age and subtype patterns. However, the association with EBV is stronger for children and older adults than young adults and stronger for the mixed cellularity subtype than the nodular sclerosis subtype most frequently diagnosed among US teens [19]. Other risk factors include family history, human immunodeficiency virus infection, autoimmune disorders, and Jewish ancestry [3]. In the past, Hodgkin lymphoma was frequently over diagnosed and, since the 1990s, there have been significant changes to the WHO classification system for lymphomas [20, 21]. More research is needed to determine what role western lifestyle and changing diagnostic accuracy and classification systems play in these incidence rate changes.

Testicular and thyroid cancers are the most rapidly increasing cancers among teens, yet the known risk factors for these cancers do not seem to offer explanation for their marked increase. Risk factors for the type of thyroid cancer that occurs most frequently in teens—papillary thyroid carcinoma—, include ionizing radiation, a mutation to the RET proto-oncogene, and family history. The only widely accepted risk factor for testicular cancer is cryptorchidism [3]. With the exception of ionizing radiation from diagnostic tests, we are unaware of evidence that any of these factors has changed dramatically over this period. If increased use of medical diagnostic x-rays is responsible for some of the increased incidence of thyroid cancer in teens, it raises the question: why are the patterns so different in females and males?

While some researchers postulate that genes and viruses are the main contributors to any observed increase in childhood cancer (including teens), other researchers argue that genes, individual susceptibility and the environment are likely to interact in such a way as to disrupt normal cell function, leading to cancer [17, 22, 23]. Evidence suggests that a substantial portion of childhood cancers, including teen cancers, may be environmentally related, and thus preventable. The President’s Cancer Panel report highlighted the increasing use of pediatric CT scans as a potential contributor to the rising rates of childhood cancer [24]. Dr. Philip Landrigan, in testimony before the President’s Cancer Panel in 2008, cited parental occupational exposures to volatile organic compounds, prenatal and early childhood exposure to pesticides, and growing up on farms or having a parent who is a pesticide applicator as part of the “emerging evidence for environmental causation of childhood cancer.” [25]. Continued surveillance of chemicals in the U.S. population, documented in the National Reports on Human Exposure to Environmental Chemicals by the U.S. Centers for Disease Control and Prevention may give a clearer picture of the amounts and trends in body burdens of potentially carcinogenic exposures in teens, as well as other age groups.

Teens present a particular opportunity for investigation of cancer etiology. In adults, latency for most solid tumors is thought to range from 10 to more than 30 years, during which a myriad of environmental exposures occur. The presumably shorter latency of teen cancer effectively reduces the list of potential environmental contributors. Increased study of this age group may lead to increased understanding of cancer etiology and identification of modifiable risk factors.

## Conclusions

Cancer in later childhood appears different in potentially important ways from early childhood cancer and deserves more study. We examined long term incidence trends for ages 15–19 using a tumor classification scheme designed for this age group and found that since 1975 overall cancer incidence increased by more than 25%, with substantial increases in thyroid cancer, testicular cancer, and NHL. The causes of these substantial increases are unknown. It is likely that these trends are the result of many different factors. A concerted research program into the causes of rising teen cancer rates is needed that can lead to interventions to alter this trend and reduce the number of teens with cancer.

## Supporting information

S1 Fig Percent contribution of histologic groups by age group. Bar graphs showing percent contribution of nine histologic cancer groups for males and females by age groups (0–14, 15–19, 20–24, 25–29, 30–34, 35–39, 40+). Adapted from Howlader et al. 2015, Tables 32.1 and 32.2.
(TIF)

S2 Fig Annual incidence trends for leukemias, acute myeloid leukemia, and lymphomas, 1975–2012. Annual rates are shown by connected points and the least squares regression line is shown as a smooth line. Regression equations and p-values for trend are also shown. SEER 9 registries include Atlanta, Connecticut, Detroit, Hawaii, Iowa, New Mexico, San Francisco-Oakland, Seattle-Puget Sound, and Utah.
(TIF)

S3 Fig Annual incidence trends for central nervous system, intracranial, intraspinal neoplasms; osseous and chondromatous neoplasms; and soft tissue sarcomas; 1975–2012. Annual rates are shown by connected points and the least squares regression line is shown as a smooth line. Regression equations and p-values for trend are also shown. SEER 9 registries include Atlanta, Connecticut, Detroit, Hawaii, Iowa, New Mexico, San Francisco-Oakland, Seattle-Puget Sound, and Utah.
(TIF)

S4 Fig Annual incidence trends for germ cell and trophoblastic neoplasms, melanoma and skin carcinomas, and carcinomas (excluding skin), 1975–2012. Annual rates are shown by connected points and the least squares regression line is shown as a smooth line. Regression equations and p-values for trend are also shown. SEER 9 registries include Atlanta, Connecticut, Detroit, Hawaii, Iowa, New Mexico, San Francisco-Oakland, Seattle-Puget Sound, and Utah.
(TIF)

S1 Table Annual number of cases by site, 1975–2012. Summary statistics for the number of cases by cancer site for the years 1975–2012.
(DOCX)
