# Supplementary figures and images for: The increasing toll of adolescent cancer incidence in the US
Source: PLoS One. 2017 Feb 24;12(2):e0172986. doi: 10.1371/journal.pone.0172986 (PMC5325567; doi:10.1371/journal.pone.0172986)

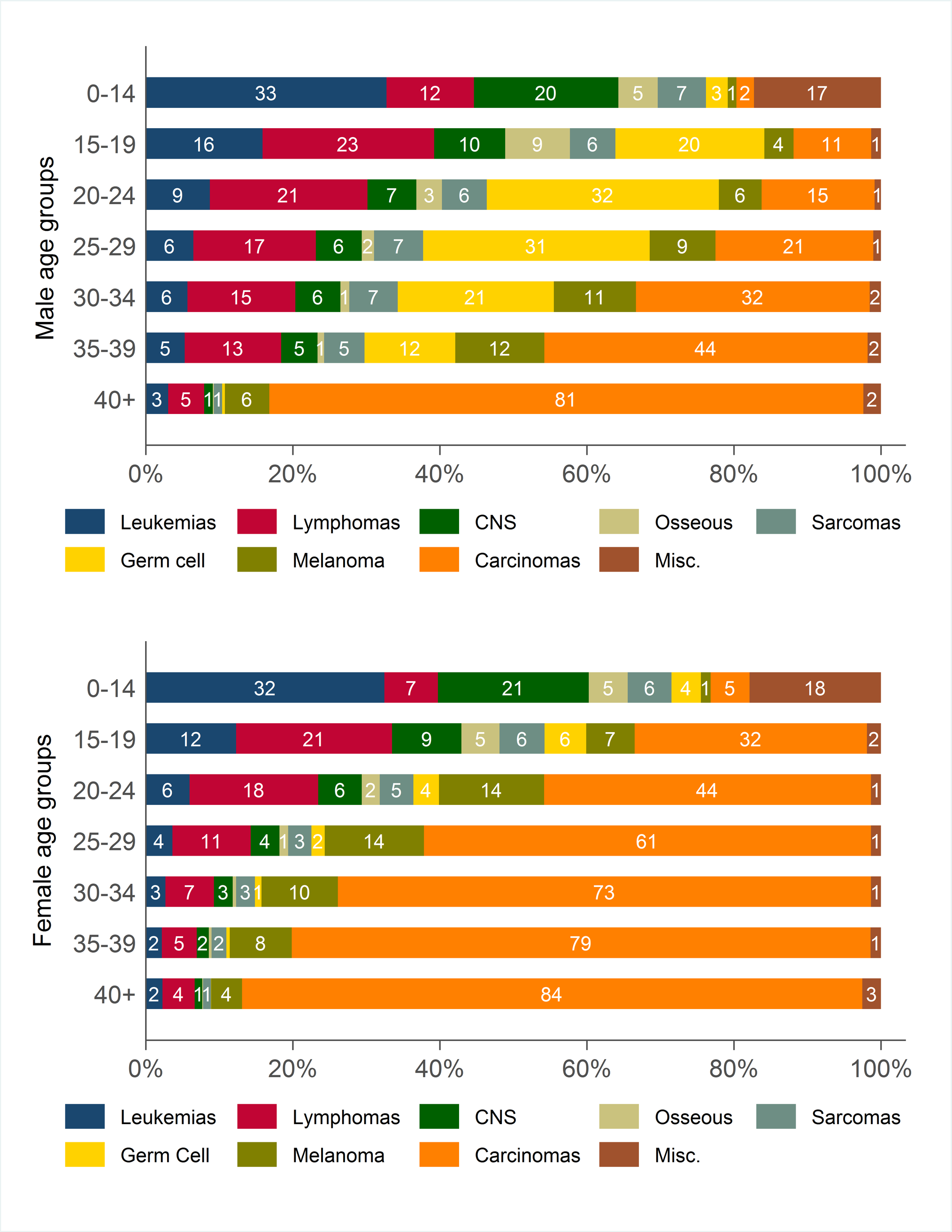

Supplement: S1 Fig — Bar graphs showing percent contribution of nine histologic cancer groups for males and females by age groups (0–14, 15–19, 20–24, 25–29, 30–34, 35–39, 40+). Adapted from Howlader et al. 2015, Tables 32.1 and 32.2. (TIF) [file pone.0172986.s001.tif]

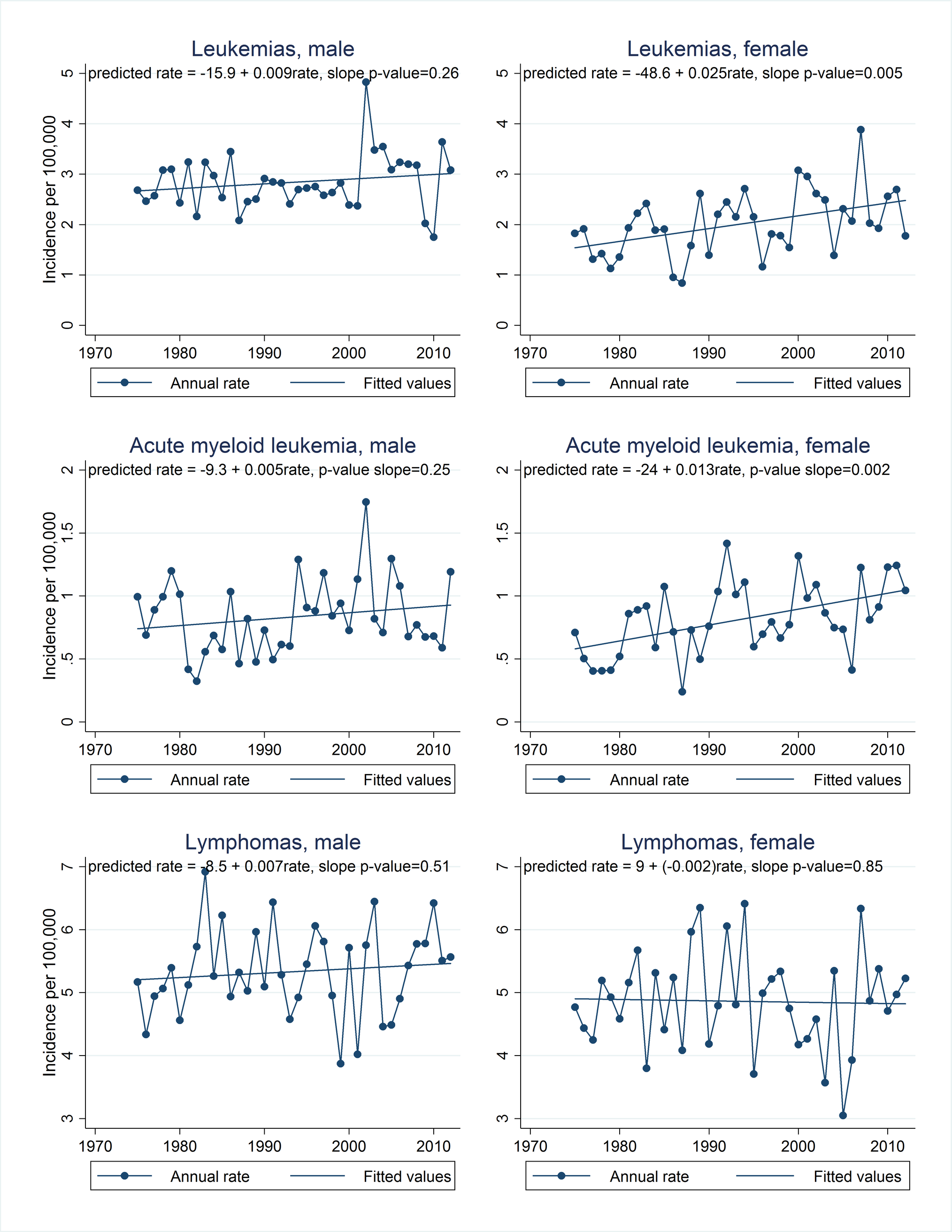

Supplement: S2 Fig — Annual rates are shown by connected points and the least squares regression line is shown as a smooth line. Regression equations and p-values for trend are also shown. SEER 9 registries include Atlanta, Connecticut, Detroit, Hawaii, Iowa, New Mexico, San Francisco-Oakland, Seattle-Puget Sound, and Utah. (TIF) [file pone.0172986.s002.tif]

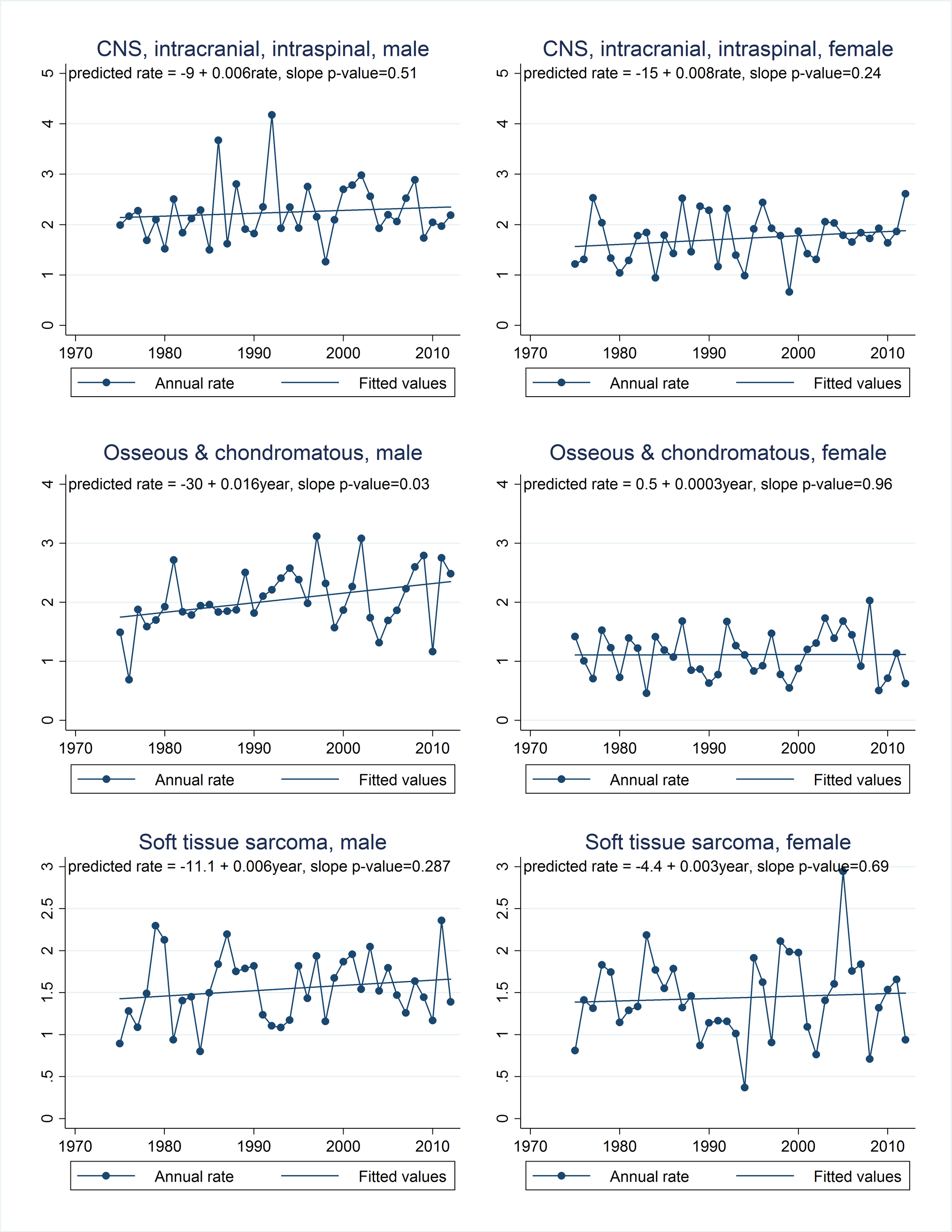

Supplement: S3 Fig — Annual rates are shown by connected points and the least squares regression line is shown as a smooth line. Regression equations and p-values for trend are also shown. SEER 9 registries include Atlanta, Connecticut, Detroit, Hawaii, Iowa, New Mexico, San Francisco-Oakland, Seattle-Puget Sound, and Utah. (TIF) [file pone.0172986.s003.tif]

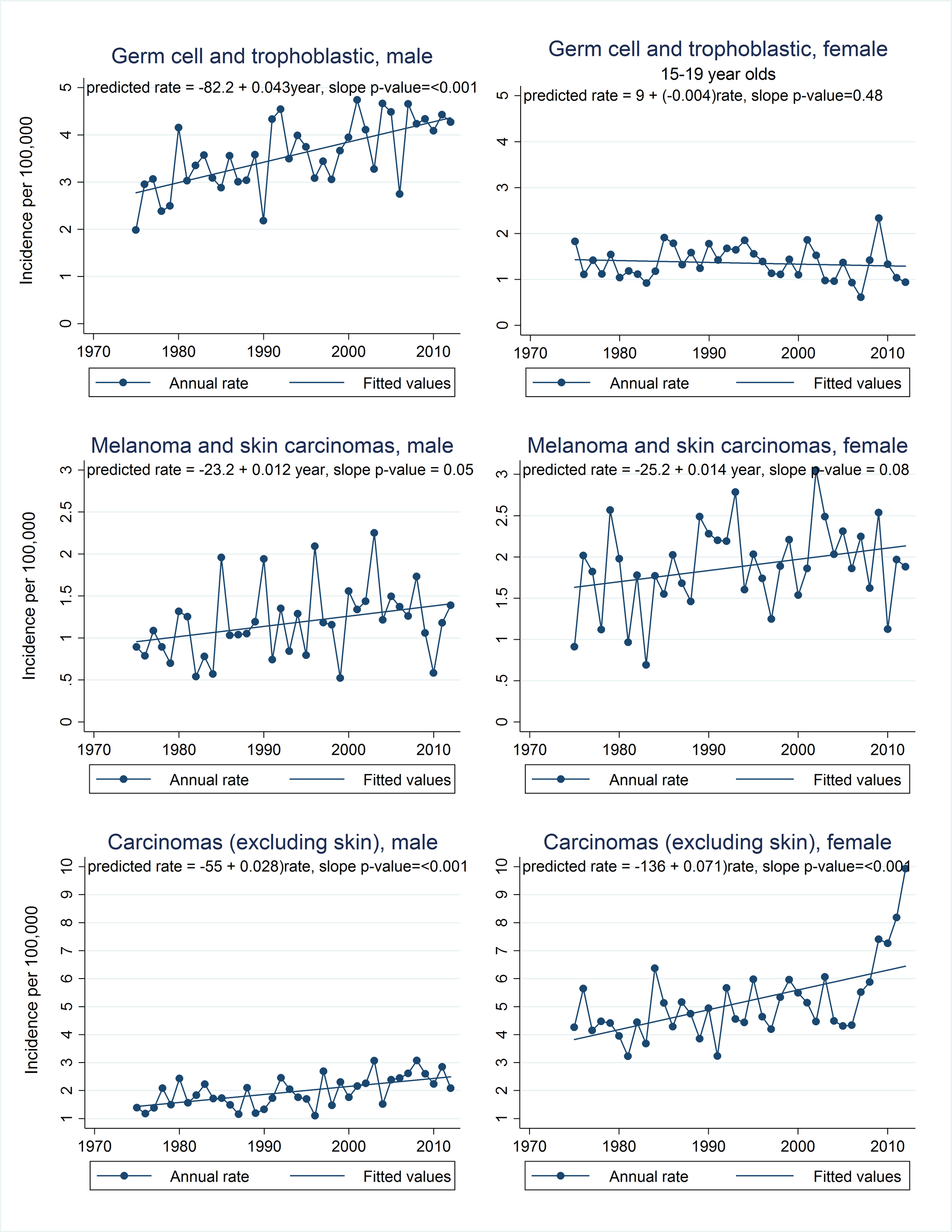

Supplement: S4 Fig — Annual rates are shown by connected points and the least squares regression line is shown as a smooth line. Regression equations and p-values for trend are also shown. SEER 9 registries include Atlanta, Connecticut, Detroit, Hawaii, Iowa, New Mexico, San Francisco-Oakland, Seattle-Puget Sound, and Utah. (TIF) [file pone.0172986.s004.tif]
