# Supplementary material for: The increasing toll of adolescent cancer incidence in the US
Source: PLoS One. 2017 Feb 24;12(2):e0172986. doi: 10.1371/journal.pone.0172986 (PMC5325567; doi:10.1371/journal.pone.0172986)
Supplement: S1 Table — Summary statistics for the number of cases by cancer site for the years 1975–2012. (DOCX) [file pone.0172986.s005.docx]

**Table S1. Annual number of incident cancer cases by site, ages 15 – 19, 1975-2012. Data from the SEER 9 Registries.**

|  | Male | | | Female | | |
| --- | --- | --- | --- | --- | --- | --- |
| **Diagnostic GROUP**/Site^a^ | Average | Minimum | Maximum | Average | Minimum | Maximum |
| **TOTAL** | 200 | 158 | 268 | 181 | 136 | 241 |
| **LEUKEMIAS** | 27 | 18 | 47 | 18 | 7 | 38 |
| Acute lymphoid leukemia | 16 | 6 | 28 | 8 | 2 | 20 |
| Acute myeloid leukemia | 8 | 3 | 17 | 7 | 2 | 12 |
| **LYMPHOMAS** | 50 | 37 | 66 | 44 | 29 | 62 |
| Non-Hodgkin lymphoma | 19 | 8 | 33 | 11 | 1 | 24 |
| Hodgkin lymphoma | 31 | 22 | 44 | 33 | 14 | 45 |
| **CNS, INTRACRANIAL, INTRASPINAL** | 21 | 12 | 34 | 16 | 6 | 25 |
| Astrocytoma | 5 | 1 | 13 | 4 | 1 | 13 |
| **OSSEOUS & CHONDROMATOUS NEOPLASMS** | 19 | 7 | 30 | 10 | 4 | 20 |
| Osteosarcoma | 10 | 2 | 20 | 5 | 1 | 9 |
| **SOFT TISSUE SARCOMAS** | 15 | 7 | 24 | 13 | 3 | 28 |
| **GERM CELL AND TROPHOBLASTIC NEOPLASMS** | 34 | 18 | 48 | 12 | 6 | 23 |
| Gonadal (Testicular/Ovarian) | 28 | 14 | 45 | 10 | 6 | 17 |
| **MELANOMA AND SKIN CARCINOMAS** | 11 | 5 | 22 | 17 | 6 | 28 |
| Melanoma | 11 | 4 | 22 | 14 | 1 | 28 |
| **CARCINOMAS (excluding skin)** | 19 | 10 | 32 | 47 | 25 | 95 |
| Thyroid | 6 | 1 | 16 | 28 | 14 | 66 |
| **MISCELLANEOUS SPECIFIED NEOPLASMS, NOS** | 4 | 1 | 11 | 4 | 1 | 10 |

^a^ Classification scheme for tumors diagnosed in adolescents and young adults (Barr 2006).
